# Editorial Notes, News and Miscellany

**Published:** 1912-12

**Authors:** 


### Editorial Notes, News and Miscellany.

The "Red Back" sends Christmas greeting to its readers and hopes they may all have a good time.

The "Red Back" also sends bills to its subscribers and solicits prompt renewals and remittances to cover arrears. Else ye editor will not "have a good time."

"Mrs. Pluto"—"Proserpine" (Eloise Shepherd) will review the paper, "How Can We Hold Our Husbands?" republished herewith, in our January number. We will have the pleasure of presenting our readers also with her picture.

Modified Milk in Infant Feeding.—The October issue of *Pediatrics* contains a paper giving a practical résumé of existing knowledge upon the modification of cow's milk as a substitute infant food. The author has made a comprehensive study of the subject from all points of view, and dwells especially upon the value of cereal decoctions in the modification of cow's milk. The practical nature of the paper will appeal to the general practitioner, and copies can doubtless be had of the author.

The American Surgical Association has appointed a committee consisting of Drs. William L. Estes, South Bethlehem, Pa., Thomas W. Huntingdon, San Francisco, Cal.; John B. Walker, New York City; Edward Martin, Philadelphia, and John B. Roberts, chairman, 313 South Seventeenth street, Philadelphia, to report on the operative and non-operative of closed and open fractures of the long bones and the value of radiography in the study of these injuries. Surgeons who have published papers relating to this subject within the last ten years will confer a favor by sending two reprints to the chairman of the committee. If no reprints are available, the titles and places of their publication are desired.

---

Christmas Is Coming!—"Save, brothers, save with care, a nickel here and a nickel there. A two-bit piece will fill the tum of some small celebrant with gum, nigger toes, peanuts and bum-bum, apples, raisins, lemon drops, popcorn, suckers, caramels, filberts, taffy, butter scotch, walnuts, figs and angel cake—save—for the Christmas belly-ache."

Now, "The frisky colt will sniff the air, and hear the whistling quail, and the festive calf will indicate the zenith with his tail. The frost will paint the forest with a deep and redder dye; the hired man will shuck the corn; the pumpkin vine will pie; the politicians will hit up their office-holding feud, and the maple tree will blush, and come out in the nude."

Now, too, "The sad-faced gobbler will address his young and tearful flock, and clip, for memory's sweet sake, a small and tear-stained lock, and then, with many sighs, will lay his head upon the block."—*The Ganderbone, St. Louis.*

---

After the Quacks and Nostrum Manufacturers.—The Post-office Department has suddenly got a move on and is after the sure cure, the lost manhood, the soothing syrup and abortifacient makers with a sharp stick. Glory hallelujah! Several hundred "doctors," he and she fellows, were arrested simultaneously in several States recently, and we will wait with bated breath, or something like that, and see what Uncle Samuel is going to do with them. It will be interesting to observe the numerous holes through which the big patent medicine makers and advertisers will escape. We have in the government service too many of the kind of men that paralyzed Dr. Wiley to hope for the enforcement of any law which will put a stop to the patent medicine evil. Mr. Hitchcock may shut the mails to them, but unless it is made a crime or misdemeanor to advertise the health-destroying nostrums, the business will continue to flourish and the ignorant to dose themselves and their babies, and suffer.

